# Métastase osseuse isolée du radius métachrone d’un cancer rectal

**DOI:** 10.11604/pamj.2013.14.161.1938

**Published:** 2013-04-25

**Authors:** Houda Eddekkaoui, Tarik Chekrine, Souha Sahraoui, Sofia Marouane, Amina Alj, Soumaya Zamiati, Mohamed Nechad, Abdellatif Benider

**Affiliations:** 1 Service de radiothérapie-oncologie, centre hospitalier Ibn Rochd, 1, quartier des Hôpitaux, 20360 Casablanca, Maroc; 2 Service central d’anatomie pathologique, centre hospitalier Ibn Rochd, 1, quartier des hôpitaux, 20360 Casablanca, Maroc; 3 Centre Petscan Rabat, 14, Angle rue Ouazzane et rue Mly Idriss Al Azhar, Hassan, Rabat, Maroc; 4 Service de chirurgie orthopédique et de traumatologie, 1, quartier des hôpitaux, 20360 Casablanca, Maroc

**Keywords:** Métastase osseuse isolée, Radius, Cancer colorectal, Isolated bone metastasis, Radius, colorectal Cancer

## Abstract

Les métastases osseuses isolées des cancers colorectaux sont très rares. Le squelette axial est habituellement le plus atteint. La localisation au niveau du radius est exceptionnelle. Nous rapportons l'observation d'une femme âgée de 60 ans avec une métastase du radius distal isolée métachrone d'un cancer du haut rectum opéré un an auparavant. La métastase a été découverte sur les examens d'imagerie et confirmée par biopsie. Une amputation a été réalisée suite à une progression de la maladie après une radiothérapie palliative. L'évolution était marquée par l'apparition de métastases pulmonaires et le décès est survenu dans un tableau de défaillance respiratoire.

## Introduction

Les métastases osseuses sont les tumeurs malignes les plus fréquentes. Elles sont particulièrement fréquentes dans les cancers du sein, de la prostate, du poumon, de la thyroïde, et du rein. Dans le cancer colorectal, elles surviennent tardivement et généralement dans le cadre de localisations métastatiques multiviscérales avec une fréquence variant selon les séries de 1 à 7% [1, 2]. L'atteinte osseuse secondaire isolée est très rare, avec une fréquence de 1,1% [3]. Nous rapportons une nouvelle observation de métastase du radius distal isolée métachrone, chez une femme opérée un an auparavant d'une tumeur du haut rectum.

## Patient et observation

Mme A.K âgée de 60 ans, a été opérée en Avril 2009 pour une tumeur du haut rectum découverte à l'occasion de rectorragies à répétition. La patiente a bénéficié d'une résection antérieure. Le bilan d'extension n'a pas objectivé de métastases à distance. L'étude anatomopathologique de la pièce opératoire a conclu à un adénocarcinome lieberkühnien infiltrant la graisse péri-rectale d'exérèse complète avec sept métastases ganglionnaires sur treize ganglions prélevés, classé pT3N2bM0. Les suites opératoires étaient simples. La patiente a reçu six cycles de chimiothérapie adjuvante associant 5- fluoro-uracile et oxaliplatine. Elle a été régulièrement suivie en consultation.

**Figure 1:**
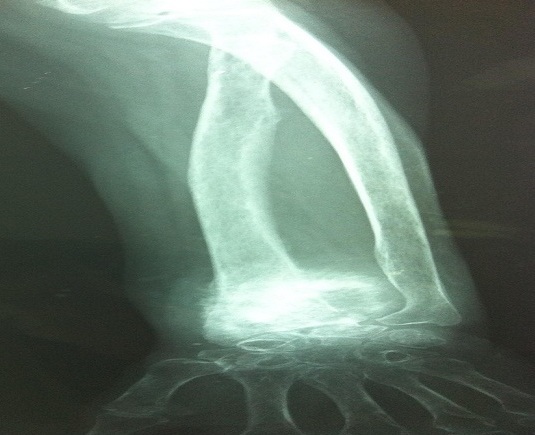
Ostéolyse de l'extrémité distale du radius gauche en radiologie standard

**Figure 2:**
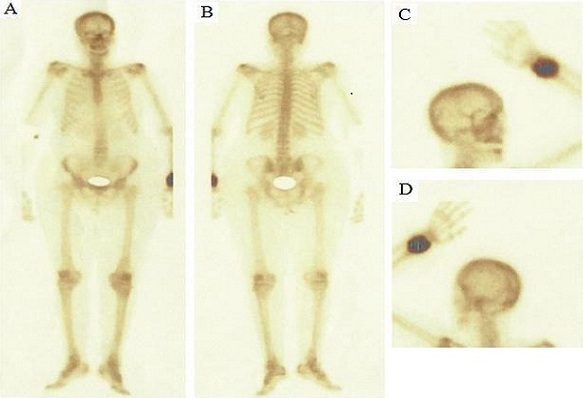
Scintigraphie osseuse montrant une hyperfixation intense unique à l'extrémité distale du radius gauche

**Figure 3:**
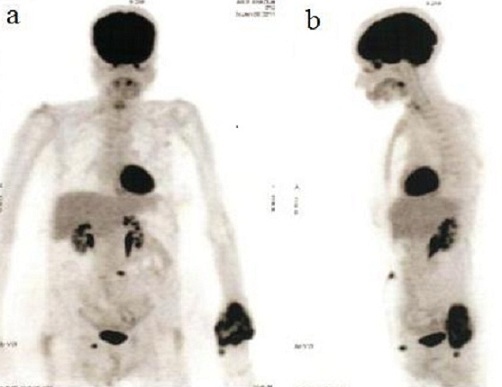
Aspect de la métastase du radius gauche à l'acquisition TEP : Foyer hypermétabolique intéressant l'extrémité distale de l'avant-bras gauche

En Avril 2010, la patiente a consulté pour une douleur intense et une tuméfaction dure du poignet gauche, sans notion de traumatisme. La radiographie standard montrait une ostéolyse de l'extrémité distale du radius gauche (Figure 1). Une tomodensitométrie de l'avant-bras gauche mettait en évidence un processus tumoral du tiers inférieur du radius avec un envahissement des parties molles. Une scintigraphie osseuse objectivait une hyperfixation intense unique du tiers inférieur du radius gauche. L'antigène carcino-embryonnaire (ACE) était à 7,33 ng/mL (N < 5 ng/mL). Une TEP-FDG complémentaire a été réalisée, à la recherche d'autres lésions à distance. Cette dernière a objectivé une volumineuse masse tumorale hyperactive de l'extrémité inférieure de l'avant-bras gauche associé à deux autres foyers hypermétaboliques suspects, le premier sous cutané sur le trajet de la cicatrice opératoire en para-ombilicale gauche et le deuxième au niveau de la région inguinale droite (Figure 2). Une biopsie de la lésion radiale gauche a confirmé le diagnostic de métastase osseuse de l'adénocarcinome rectal déjà traité (Figure 3). Devant le doute sur la nature métastatique des deux foyers hypermétaboliques para-ombilicale gauche et inguinale droite, une exérèse des deux lésions a été réalisée, ne montrant pas de malignité. La patiente a reçu une radiothérapie antalgique sur le poignet gauche à la dose de 20 Gy à raison de 5 fractions de 4 Gy suivie de trois cures de chimiothérapie associant capécitabine et irinotécan. Devant l'aggravation de la symptomatologie clinique, l'augmentation de volume de la métastase du radius gauche et après discussion du dossier en réunion de concertation pluridisciplinaire, une amputation de l'avant-bras a été réalisée. L'évolution était marquée par l'altération de l'état général et l'apparition de métastases pulmonaires. La patiente décédait dans un tableau de défaillance respiratoire un an après le diagnostic de la métastase du radius.

## Discussion

Les métastases osseuses des cancers colorectaux sont moins fréquentes que les métastases hépatiques et pulmonaires. Leur fréquence varie de 1 à 7% [1, 2] et peut atteindre jusqu'à 20% dans des séries autopsiques [1]. Cette atteinte osseuse secondaire est le plus souvent d'apparition tardive dans l′histoire naturelle d′un cancer colorectal métastatique déjà connu [3, 4].

L'atteinte osseuse isolée est encore plus rare [2, 3]. Curling en 1870, a décrit le premier cas de métastase osseuse du radius d'un cancer du rectum [5]. Nous rapportons, à notre connaissance le deuxième cas après celui de Curling. Plusieurs autres sites anatomiques inhabituels : tibia, mandibule, omoplate, os de la main et du pied, ont été rapportés. Les cancers rectaux semblent par ailleurs davantage ostéophiles que les cancers coliques [2]. Cette différence de fréquence semble être corrélée à un mécanisme vasculaire par la proximité anatomique du rectum du plexus veineux paravertébral [2]. Aussi, le carcinome à cellules en bague à chaton présente une incidence élevée de métastases osseuses par rapport aux autres types histologiques [2, 4].

Pour la distribution topographique des métastases osseuses, le squelette axial (rachis et bassin) est le plus souvent atteint. Ceci s'explique par l'invasion osseuse qui se fait par voie hématogène essentiellement via le plexus veineux paravertébral de Baston [6] après invasion directe des veines par la tumeur. Cependant, tous les sites osseux peuvent être envahis : os longs et particulièrement le fémur, le crâne, les côtes, la ceinture scapulaire [2]. Les communications entre les veines lombaires et le système veineux ilio-fémoral expliquent l'atteinte des membres inférieurs aussi bien pour les tumeurs colorectales que pour les tumeurs vésicales [7].

Cliniquement, la symptomatologie est non spécifique: tuméfaction osseuse, douleur, compression nerveuse périphérique, compression médullaire, hypercalcémie. Les fractures spontanées semblent rares [8]. Le délai d'apparition par rapport à la découverte de la tumeur primitive varie de 10 jours et 14 ans [3]. La scintigraphie osseuse reste l'examen le plus efficace pour la détection précoce des métastases osseuses [9]. Talbot et al. [10] ont trouvé que les patients présentant des métastases osseuses symptomatiques ont été diagnostiqués avec une médiane de 21 mois après l′exérèse chirurgicale de la tumeur primitive. Dans le cas que nous rapportons, la métastase du radius a été découverte 12 mois après l'exérèse de la tumeur rectale.

Radiologiquement, les métastases osseuses de cancer colorectal sont généralement ostéolytiques. L'aspect mixte ostéolytique et ostéoblastique et l'aspect pseudosarcomatoïde avec ossification des parties molles ne sont pas rares. [9]. En raison de l'aspect ostéolytique et du caractère isolée, le diagnostic d′une lésion maligne primitive de l′os doit être suspecté. Un scanner est utile pour démontrer l'extension excentrique de la corticale qui est généralement observé dans les métastases. Cependant, une biopsie osseuse est obligatoire pour confirmer le diagnostic [8].

Kanthan et al. [3], sur une série de 355 patients ayant des métastases osseuses de cancer colorectal, dont 60 étaient isolées et 295 associées à des métastases viscérales, les taux de survie à cinq ans étaient respectivement de 38% et 16%. Par contre, la survie à 10 ans n′était pas significativement différente entre les deux groupes.

Nozue et al [4], ont retrouvé que la survie après l′apparition des métastases osseuses était très faible, avec une médiane de survie d′environ 5 mois et un taux de survie à un an de 20%. Seuls deux cas de métastases osseuses isolées ont vécu plus d'un an. Ce mauvais pronostic conditionne le traitement. Il est donc palliatif, il vise à soulager la douleur et à améliorer la qualité de vie. La radiothérapie est préférée par plusieurs auteurs, du fait de l'impact psychologique d'une amputation, considérée par le patient comme une mutilation.

## Conclusion

Les métastases osseuses isolées des cancers colorectaux sont rares. Une douleur osseuse et /ou une anomalie radiologique devraient alerter les cliniciens sur la possibilité d'une métastase osseuse même en l'absence d'autres sites métastatiques à distance tels que le foie et les poumons. Des localisations inhabituelles comme le cas que nous décrivons sont possibles. Un diagnostic précoce est important pour améliorer la qualité de vie des patients.
